# High-dose methadone detoxification in a hospital setting: A case report

**DOI:** 10.1192/j.eurpsy.2026.10879

**Published:** 2026-07-31

**Authors:** J. Gimillo Bonaque, C. D. Mayoral, M. S. Molina

**Affiliations:** Universitario Príncipe de Asturias, Hospital, Alcalá de Henares, Spain

## Abstract

**Introduction:**

Present the case of a 37-year-old patient with a diagnosis of antisocial personality disorder who has a long-standing heroin addiction and an occasional use of cannabis and alcohol. The patient required admission to the Gastroenterology department for a stomach issue, during which he was administered 100 mg of Methadone daily. Upon discharge, he was no longer using heroin and requested, few weeks later, a scheduled admission to a detoxification unit from his outpatient clinic for a complete Methadone withdrawal. He did not accept buprenorphine substitution treatment.

**Objectives:**

The objective is to review the literature regarding methadone dose tapering and how to manage it during hospitalization. Patient rejected current guidelines initially recommendation (partial agonist like Buprenorphine).

**Methods:**

Narrative presentation of the proposed case and a comparison of its management with the current scientific literature.

**Results:**

The usual protocol for Methadone detoxification has been a very slow, gradual reduction of the agonist dose on an outpatient basis, as has been traditionally recommended, with a weekly decrease of 3%. In general, a gradual reduction of a maximum of 10% of the dose per day is recommended, although it is advisable to do so more slowly (1-5 mg/week).

We opted to discontinue methadone from the first day and use alpha-2 agonists, which had demonstrated their usefulness in treating withdrawal syndrome in both inpatient and outpatient settings. The use of Clonidine plays a key role in reducing the noradrenergic hyperactivity that occurs during opioid withdrawal syndrome (OWS) and in blocking the symptoms precipitated by Naltrexone. We did not achieve optimal blood pressure levels, although there were no severe episodes of hypotension. The patient complained of a certain general malaise and restlessness during the first few days, which we managed with medium doses of Gabapentin. Naltrexone is administered starting on the seventh day of admission and Methadone abstinence, after verifying abstinence with a Naloxone challenge test. Discharge occurs between the eighth and tenth day of admission detoxification.

The literature recommends Clonidine doses of 0.15 to 0.45 mg per day during hospitalization, with a gradual increase and close monitoring of blood pressure to prevent significant fluctuations in these values and typical withdrawal symptoms like dizziness, nausea, or vomiting. Additionally, a reasonable use of Clonazepam (1 to 6 mg) is recommended to assist with this management.

**Conclusions:**

We emphasize the importance of systematically monitoring the patient’s vital signs and providing adequate rehydration for better management of withdrawal symptoms. There is no need for excessive use of benzodiazepines; instead, the patient should be psychoeducated about opioid withdrawal syndrome before admission.

**Disclosure of Interest:**

None Declared

